# Systemic delivery of large-scale manufactured Wharton’s Jelly mesenchymal stem cell-derived extracellular vesicles improves cardiac function after myocardial infarction

**DOI:** 10.20517/jca.2021.21

**Published:** 2022-01-05

**Authors:** Michael A. Bellio, Rosemeire M. Kanashiro-Takeuchi, Lauro Takeuchi, Shathiyah Kulandavelu, Yee-Shuan Lee, Wayne Balkan, Karen C. Young, Joshua M. Hare, Aisha Khan

**Affiliations:** 1 Interdisciplinary Stem Cell Institute, University of Miami Miller School of Medicine, Miami, FL 33136, USA; 2 Department of Molecular and Cellular Pharmacology, University of Miami Miller School of Medicine, Miami, FL 33136, USA; 3 Department of Pediatrics, University of Miami Miller School of Medicine, Miami, FL 33136, USA; 4 Department of Medicine, University of Miami Miller School of Medicine, Miami, FL 33136, USA; 5 Division of Neonatology, University of Miami Miller School of Medicine, Miami, FL 33136, USA

**Keywords:** Extracellular vesicles, Wharton’s Jelly, mesenchymal stem cells, manufacturing, myocardial infarction

## Abstract

**Introduction:**

Cardiovascular disease and myocardial infarction are leading causes of morbidity and mortality in aged populations. Mesenchymal stem cell (MSC)-derived extracellular vesicles (EVs) are under evaluation as a therapeutic option for the treatment of myocardial infarction.

**Aim:**

This study aimed to develop a large-scale manufacturing procedure to harvest clinical-grade EVs required for the translation of EVs to the clinic.

**Methods and Results:**

We compared the efficiency of large scale MSC-derived EV production and characterized EV miRNA cargo using the Quantum bioreactor with either fetal bovine serum or human platelet lysate (PLT)-containing expansion media. We tested the potency of the EV products in a murine model of acute myocardial infarction. Our results demonstrate an advantage of the Quantum bioreactor as a large-scale platform for EV production using PLT media; however, both media produced EVs with similar effects *in vivo.* The systemic delivery of EV products improved cardiac function following myocardial infarctions as indicated by a significant improvement in ejection fraction as well as parameters of cardiac performance, afterload, contractility and lusitropy.

**Conclusion:**

These findings have important implications for scale-up strategies of EVs and will facilitate clinical trials for their clinical evaluation.

## One sentence summary

Large scale manufacturing of MSC-derived EVs is feasible and when delivered systemically, improves cardiac function after myocardial infarction.

## INTRODUCTION

Cardiovascular aging is associated with molecular and physiological changes that result in the increased incidence of cardiovascular diseases such as hypertension, atherosclerosis, and myocardial infarction (MI)^[1]^. As the aged population continues to grow in number, novel therapies aimed at repairing the damaged cardiovascular system are required to provide effective medical solutions. Mesenchymal stem cell (MSC) derived extracellular vesicles (EVs) are presently under evaluation as a potential regenerative medicine approach. MSCs are found in many human organs and have been safely tested in clinical trials using an allogeneic strategy^[2–4]^. Phase I/II clinical trials such as POSEIDON^[5]^, POSEIDON-DCM^[6]^, and TRIDENT^[7]^ have demonstrated the safety and potential efficacy of allogeneic MSCs for the treatment of ischemic and non-ischemic cardiomyopathy. In terms of mechanism of action, there is growing awareness that MSCs release EVs and other growth factors that exert paracrine effects. Given the ability to purify EVs, it seems logical to test them for their ability to exert many of the actions of the parent cells^[8,9]^.

Pre-clinical data collected across multiple regenerative medicine models suggests that a primary mechanism of MSC-induced regeneration is mediated by the release of EVs into the extracellular space^[10,11]^. EVs are important for cell-to-cell communication and produce a paracrine effect when used as a therapeutic agent. MSC-derived EVs promote tissue recovery through restoring tissue homeostasis, inhibiting inflammation, and promoting angiogenesis^[12–15]^. Isolation of MSC-derived EVs from *in vitro* conditioned media has demonstrated important cardiac reparative effects, and thus further exploration is warranted^[16]^.

Despite promising pre-clinical data, several challenges impede the translation of EVs into cardiovascular clinics. These challenges include establishing advanced characterization methods for documented reproducibility, the large-scale production of EV concentrated fluid, and the large-scale isolation and processing of clinical-grade, FDA-compliant EV products for Investigational New Drug enabling studies or clinical application^[17]^. Traditional EV production techniques are often limited due to the need for repeated and lengthy manufacturing protocols and characterization of each product lot. Furthermore, the need for repetitive lot productions of cell source products can lead to greater batch-to-batch variability and the need for repeated manufacturing runs and testing time.

Wharton’s Jelly (WJ), derived from umbilical cord tissue, is a good source of MSCs due to non-invasive collection techniques and the natural pool of fetal sourced cell types. Therefore, we developed a large-scale manufacturing protocol utilizing Wharton’s Jelly-derived MSCs (WJMSCs) to efficiently streamline the manufacture of EVs for research and clinical application.

During the development of our large-scale production methodology, we investigated the effect of cell culture media composition on the efficiency and characteristics of EV production. Fetal bovine serum (FBS) is commonly used as a growth media supplement for the expansion of MSCs^[18]^. However, due to the FDA’s concerns involving the use of animal-derived products, human platelet lysate (PLT) has become a commonly used alternative for clinical cell expansion. PLT is a safe and effective alternative to FBS that retains MSC phenotype and promotes cell proliferation and expansion potential^[19]^.

MSC-derived EVs have a well-documented track record of promoting cardiac protection and restoring cardiac function in various models of MI^[20]^. In these models, EVs are administered immediately after cardiac injury and administered by intra-myocardial injection or by tail vein^[21,22]^. The immediate delivery of EVs to an injury site speaks to the cardioprotective effects of MSC-derived EVs and the therapeutic value of reducing or preventing the initial damage caused by MI. However, these models have yet to explore EVs as a therapeutic option after established MI injury and the subsequent long-term effects that the EV products have on cardiac function.

In this study, we used manufacturing data and *in vivo* analysis to determine the optimal conditions to produce large quantities of EVs for use as a therapeutic for MI. We hypothesized that PLT-supplemented media would be superior for the large-scale expansion and production of EVs and that systemic delivery of WJMSC-derived EVs would improve cardiac function and reduce cardiac remodeling compared to placebo.

## METHODS

### WJMSC isolation and expansion

This study protocol and written informed consent were reviewed and approved by the Institutional Review Board of the University of Miami (IRB number 20100986). WJ was isolated from human umbilical cord obtained from healthy and full-term infants. WJ was cut into small pieces, placed into several Petri dishes with a minimum medium to allow for attachment. The dishes were placed into a 37 °C incubator with 5% CO_2_. The growth medium was composed of 20% FBS and 1% penicillin/streptomycin in α-MEM or 5% PLT max (Millcreek), heparin, and 1% penicillin/streptomycin in α-MEM. Three umbilical cords were used for these experiments, where each cord was split into FBS and PLT culture after WJ isolation. One day after plating, a small amount of medium was added to the WJ to ensure the WJ pieces were attached securely. The medium was replenished every three to four days until the colonies were 90% confluent. Once confluent, the cells were rinsed once with phosphate-buffered saline (PBS) and then incubated with TrypLE (Thermofisher) for 5 min at room temperature, and the TrypLE was neutralized with medium and the cell suspension centrifuged at 200 x*g* for 10 min. The cells were counted, and 0.5 to 1 × 10^6^ cells were seeded into each T-175 flask. The cells in these new flasks were designated as passage 1. Fresh medium was replenished every three to four days until the cells reached 90% confluency. When passage 1 cells reached confluency, the cells were harvested and cryopreserved at 2 to 5 × 10^6^ cells/mL of cryopreservation medium. The cryopreservation medium used was CryoStor CS_10_ (BioLife Solutions). Flow Cytometry analysis with CD_31_, CD_45_, CD_105_, CD_90_, and isotype control was performed on P_2_ WJMSC expanded in both PLT and FBS to confirm their MSC origin [Supplementary Figure 1].

### Quantum bioreactor MSC expansion and EV isolation

The Quantum bioreactors (Terumo BCT) were primed with PBS and coated with 5 mg of human fibronectin (Corning) diluted in 100 mL of PBS. Fibronectin coating was performed for a minimum of 4 h, followed by a systemic washout with expansion media supplement with either 20% FBS or 5% PLT. After expansion media exchange, 30.0 × 10^6^ WJMSCs were loaded and allowed to attach for 24 h. Once cell attachment was completed, cell expansion was performed by increasing the daily media input feeding rate to compensate for the growing number of cells. Samples of the outer loop media were collected daily to test for lactate production, which was used to estimate the total number of growing cells. The estimation and prediction of cell numbers were determined by daily lactate production readings, media flow rate, and excel sheet calculations and formulas provided by Terumo BCT. After peak expansion was obtained, expansion media was washed away with inner and outer loop washouts with PBS. After completion of the washout cycle, the media was replaced with α-MEM without FBS or PLT. Therefore, EV manufacturing was performed without the presence of any protein supplement. Media conditioning was then allowed to continue for 120 h, and inner loop outlet waste media was collected daily for EV isolation. Every 24 h, 200 mL of conditioned media was collected from the Quantum bioreactor and subjected to sequential centrifugation at 2000 x*g* for 30 min followed by 20,000 x*g* for 30 min to remove large EVs prior to ultracentrifugation. Ultracentrifugation (Beckman Coulter) was performed at 100,000 x*g* for 70 min using a Ti70 rotor to pellet the remaining EVs. EV pellets were resuspended in 4-5 mL of saline and frozen at −80 °C. Samples were resuspended in sterile saline to make the final product ready for injection.

### Nanosight analysis

Nanosight nanoparticle analysis was performed on the final EV products using the Nanosight NS300 instrument (Malvern Panalaytical) and NTA 3.3 Dev Build 3.3.104 software. Mean concentrations and mode size were determined from 5 videos taken of 1 sample analyzed at a 1:1000 dilution, pump speed 30, video length 30 s.

### EV flow cytometry analysis

Flow cytometry of EV nanoparticles was completed using Exosome-Human CD63 Isolation/Detection Reagent (Thermofisher) according to the manufacturer’s instructions. EVs collected at 48 h and 120 h were analyzed as representative examples for the detection of CD63 and CD81, exosome surface markers. Precipitated CD63 particles were stained with CD63 antibody, CD81 antibody, or FITC and APC isotype controls. Samples were analyzed on a Cytoflex flow cytometer (Beckman Coulter).

### miRNA isolation, sequencing, and bioinformatics

WJMSC-derived EVs isolated from three donors were manufactured from FBS- and PLT-expanded cells, and processed for miRNA isolation and sequencing in triplicate. miRNA extraction and sequencing were conducted by QIAGEN Genomic Services. RNA was isolated using the exoRNeasy Kit Part II (Qiagen) and automated on the QIAcube. 700 μL of QIAzol was added to the EVs and then continued with the 2nd part of the exoRNeasy midi kit for RNA purification as per manufacturer’s protocol. RNA was extracted from the samples, including RNA spike-ins in the purification step to allow monitoring of the RNA extraction efficiency. Each RNA sample was successfully reverse transcribed (RT) into cDNA and tested for the expression of 5 miRNAs and three synthetic spike-ins. RNA was reversely transcribed in 10 μL reactions using the miRCURY LNA RT Kit (Qiagen). cDNA was diluted 100× and assayed in 10 μL PCR reactions using the miRCURY LNA SYBR Green PCR Kit (Qiagen). The amplification was performed in a LightCycler 480 System (Roche) in 384-well plates. The amplification curves were analyzed using Roche LC software, both for determination of Cq (by the 2nd derivative method) and for melting curve analysis. The raw data was extracted from the Roche LC software. The evaluation of expression levels was performed based on raw Cq values. The library preparation was done using the QIAseq miRNA Library Kit (QIAGEN). A total of 5 μL total RNA was converted into miRNA NGS libraries. Adapters containing unique molecular identifiers were ligated to the RNA. Then RNA was converted to cDNA. The cDNA was amplified using PCR (22 cycles), and during the PCR, indices were added. After PCR, the samples were purified. Library preparation QC was performed using either Bioanalyzer 2100 (Agilent) or TapeStation 4200 (Agilent). The library pool was then sequenced on a NextSeq500 sequencing instrument according to the manufacturer’s instructions. Raw data was de-multiplexed, and FASTQ files for each sample were generated using the bcl2fastq software (Illumina Inc.). FASTQ data were checked using the FastQC tool. FASTQ data was processed in QIAGEN’s Omicsoft Array Suite (version 10.1.1.14). Following the preprocessing of the reads, reads were aligned to the genome using OSA. All statistical analysis and miRNA identification was performed by the QIAGEN Genomic Services. Bioinformatics was completed using the IPA Knowledge Base software (version 01-16), and mature miRNA and mRNA target relationships were determined. All gene targeting was limited to relationships with experimental observed findings. mRNA target lists were used for Bioprofiler analysis to identify myocardial infarction associated genes. Vinny 2.1 was used to create vin diagram comparison charts.

### Murine myocardial infarction model and EV treatment

All animal experiments were performed in accordance with the protocol approved by the University of Miami IACUC. Three-month-old male *C57BL6J* mice were induced and anesthetized with isoflurane (2%-3%) inhalation. Electrocardiogram, body temperature, and heart rate were monitored throughout the procedure. MI was induced through the permanent ligation of the left coronary artery with a 7-0 Prolene suture as previously described with minor modifications^[23]^. MI was confirmed by ST elevation and myocardium blanching during the procedure. Extended-release buprenorphine was given pre-operatively (0.5-1 mg/kg). Echocardiography at day 7, post-surgery was performed before randomization to one of three treatment groups: placebo (*n* = 14), EV-PLT (EVs isolated in serum-free media from PLT-expanded WJMSCs, *n* = 14), or EV-FBS (EVs isolated in serum-free media from FBS-expanded WJMSCs, *n* = 10).

EV products were delivered by intra-jugular injection at a dose of 1 × 10^10^ particles in 100 μL of saline, while 100 μL of saline only was used as the placebo. Treatments began 1 week after the MI and were repeated weekly; the animals received 6 total doses of EVs or placebo and were monitored for 8 weeks [Supplementary Figure 2]. To perform the weekly injections, the animals were anesthetized with isoflurane (1%-3%), and the area over the right and/or left jugular was shaved to expose the external jugular veins. The 29G needle (BD) was inserted through the pectoral muscle below the sternoclavicular junction. After the injection, gentle pressure to the injection site was applied to prevent bleeding, and the animal was allowed to recover before its return to the cage. Investigators administering the products were blinded to the treatment.

### Echocardiography

Cardiac function [EF, cardiac output (CO), and stroke volume (SV)] were calculated using Vevo-2100 imaging system (Visual Sonics Inc.) at baseline, 1-, 4- and 8-week post-MI as previously described with minor modifications^[23,24]^. Briefly, mice were anesthetized by inhalation with 1%-3% isoflurane while heart rates and body temperatures were continuously monitored. Cardiac morphology and function were analyzed using an automated left ventricular (LV) analysis software (AutoLV, Visual Sonics Inc.) at baseline, 1-, 4-, and 8-week post-MI. All assessments of imaging and data analysis were performed by the investigator blinded to the treatment.

### Hemodynamic study

Pressure-volume data were obtained from steady-state and inferior vena cava occlusion using a Power Lab data acquisition system (ADInstruments, Denver, CO) as previously described^[23,24]^. A microtip pressure-volume catheter (SPR 839, Millar Instruments) was introduced into the surgically exposed right carotid artery and advanced into the left ventricle. Hemodynamic data were analyzed offline by investigators blinded to treatment groups using Lab Chart 8.1.5 Pro Software (ADInstruments). The volume calibration was performed using end-diastolic volume and end-systolic volume derived from echocardiographic measurements.

### MI quantification

Masson’s trichrome-stained sections were used to assess infarct size. Histological sections were scanned with a digital pathology slide scanner (PathScan Enabler IV, Meyer Instruments), and infarct size was measured using MIQuant software as described^[25]^. The infarcted LV wall was calculated by the midline length measurement (calculated by dividing the midline length of the infarcted LV wall by the midline length of the total LV wall) as previously validated by Nascimento *et al.*^[25]^. Only regions with infarct in > 50% of the whole thickness of the myocardium were considered for infarct midline^[26]^.

### Statistics

Statistics were completed using Prism 8 (Version 8.4.2). WJMSC EV expansion data was analyzed between-and within-group using two-way ANOVA and multiple comparisons test with Tukey. *P*-values less than 0.05 were significant. Two group column statistics were performed using a 2-tail *t*-test. Echocardiography and pressure-volume (PV) loop data were analyzed using one-way ANOVA with Tukey’s multiple comparison test. Infarct size was analyzed by one-way ANOVA comparing the mean of each group to placebo with Dunnett multiple comparison testing. Averaged data with error bars include ± the standard error of the mean.

## RESULTS

### Large scale expansion efficiently produced WJMSC-derived EVs in both FBS and PLT

**Figure 1.**
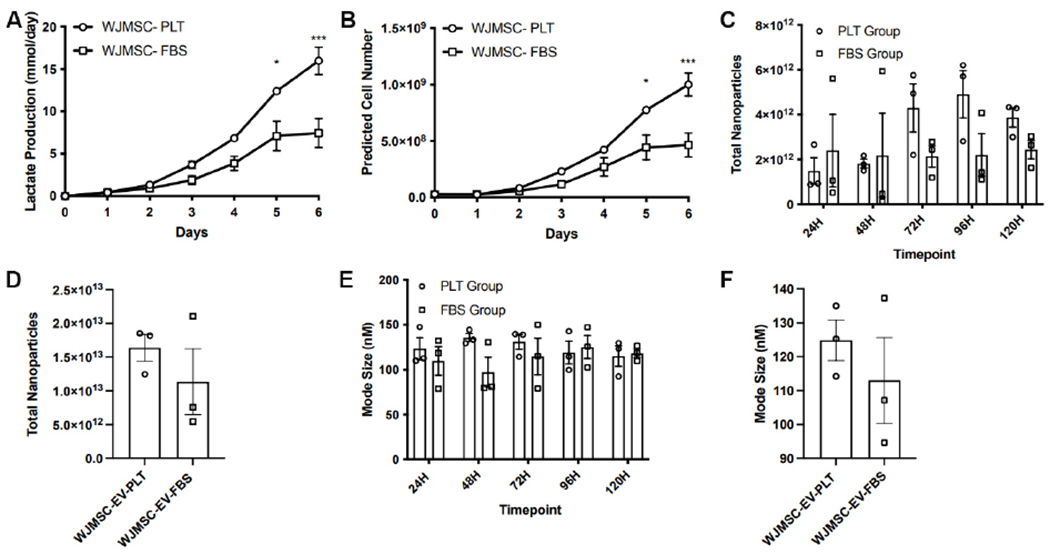
WJMSC expansion and EV manufacturing in the quantum bioreactor. (A) Recorded values of the daily lactate production during WJMSC expansion. (B) The daily prediction of cell numbers within the bioreactor. (C) The average number of EVs isolated at each collection point. (D) The average total number of EVs collected in a single bioreactor run. (E) The average mode size of the EV harvest at each collection time point. (F) The average mode size of the EVs isolated from a single bioreactor run. *P < 0.05, ***P < 0.001. WJMSC: Wharton’s Jelly-derived mesenchymal stem cell; EVs: extracellular vesicles; PLT: human platelet lysate; FBS: fetal bovine serum.

WJMSC expansion in the Quantum bioreactors was completed in six to seven days. Throughout this expansion time, daily lactate measurements were recorded to assess cell growth. Maximum lactate production was achieved after six days of expansion, indicating peak cell confluency. Comparative analysis of lactate production and the calculated predicted cell yield between PLT or FBS demonstrated a more efficient cell expansion of WJMSC when expanded in media with PLT. Significantly higher lactate measurements in the PLT expansion at day 6 (16.0 ± 1.6 mmol/day in PLT *vs*. 7.43 ±1.7 mmol/day in FBS, *P*-value < 0.001) indicates a superior cell expansion [Figure 1A]. The predicted cell number, which is based on daily lactate production, further demonstrated the improved cell expansion using the PLT media compared to FBS [Figure 1B].

After maximum cell expansion was obtained, PLT and FBS containing medias were replaced with serum-free media, and EV harvest and isolation were performed every 24 h for 120 h. No significant difference was observed in the number of particles harvested daily in the FBS or PLT groups [Figure 1C]. Similarly, there was no significant difference between the total number of harvested particles, 1.64 ± 0.2 × 10^13^ and 1.13 ± 0.5 × 10^13^ nanoparticles for the PLT and FBS group, respectively [Figure 1D]. Furthermore, nanoparticle tracking analysis of the harvested products revealed no difference in the mode particle size of the EVs from each group, an average mode size of 125 ± 4.4 nm and 113 ± 6.2 nm for the PLT and FBS group, respectively [Figure 1E and F].

**Figure 2.**
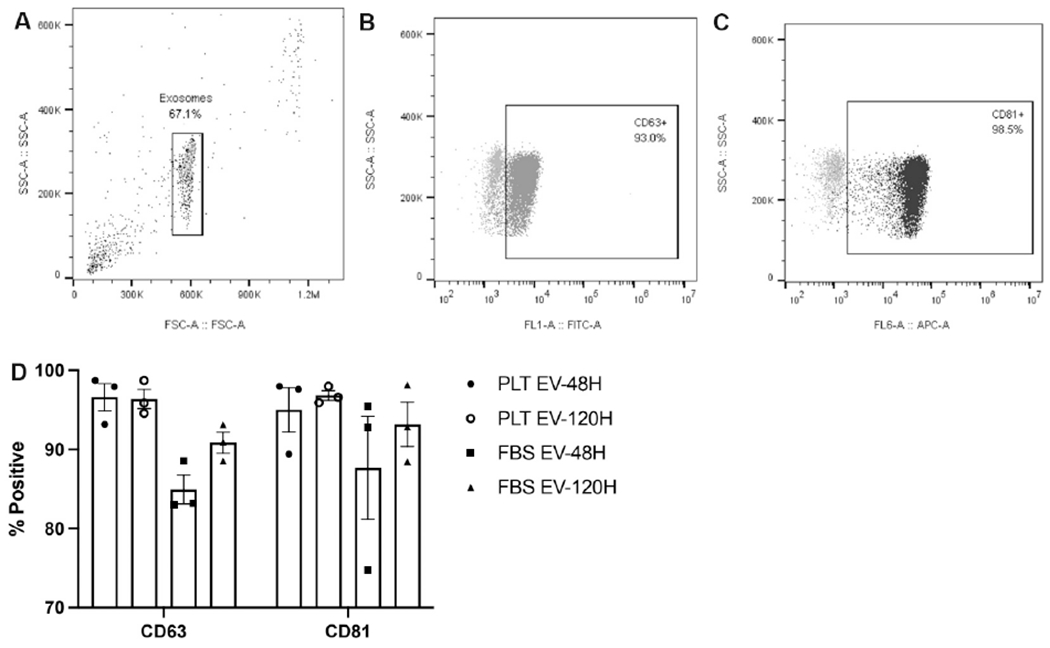
Flow cytometry analysis of the EV products. (A) Representative scatter plots showing the analyzed EV population. (B) Positive expression of FITC+/CD63+ EVs (dark gray) compared to isotype control (light gray). (C) Positive expression of APC+/CD81+ EVs (black) compared to isotype control (light gray). (D) The calculated CD63 and CD81 positive expression of the EV products collected at the 48 and 120 h collection time points. EVs: Extracellular vesicles; PLT: human platelet lysate; FBS: fetal bovine serum.

EV products collected at 48 and 120 h were analyzed by flow cytometry to confirm the presence of EV markers CD63 and CD81. There was no observed difference in the expression of these exosome markers between FBS- and PLT-expanded products [Figure 2].

### Comparative analysis of FBS *vs.* PLT produced EV cargo

**Figure 3.**
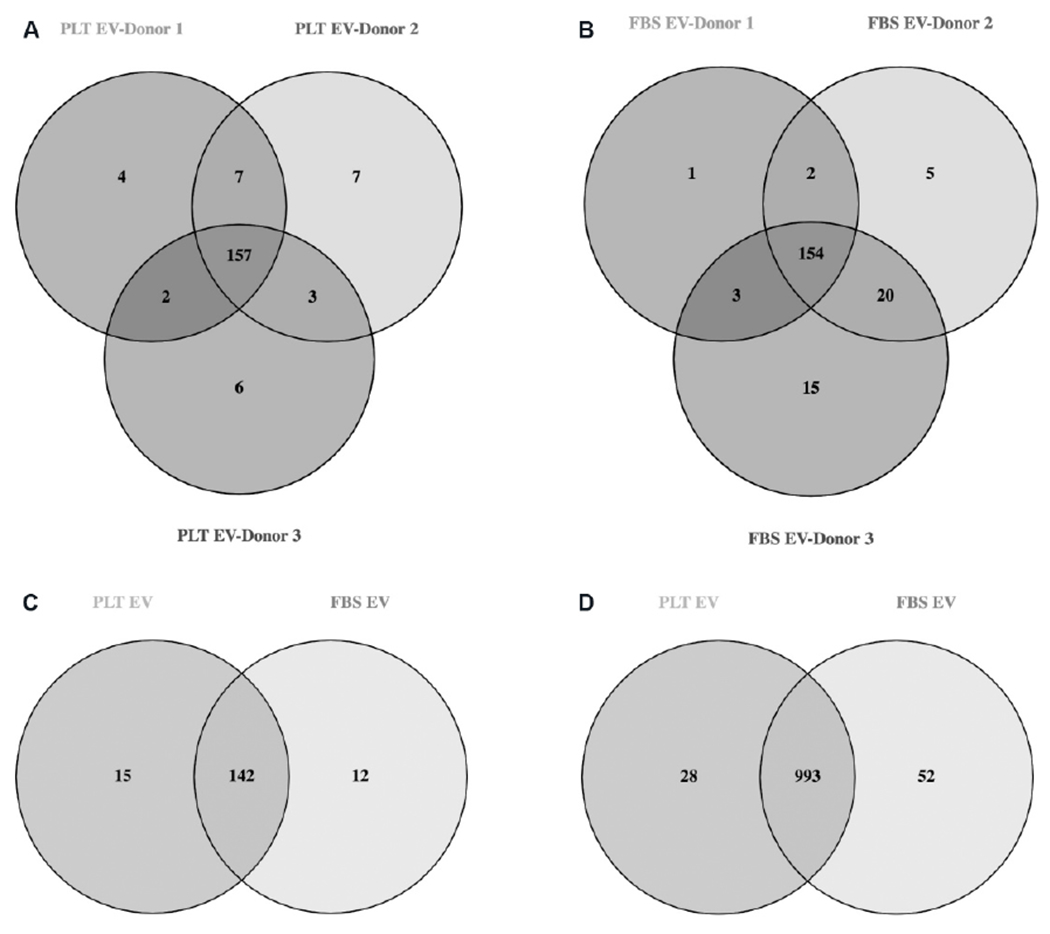
Identification and differential expression analysis of EV miRNA cargo. (A) The identification of commonly expressed miRNA between 3 different PLT-EV products manufactured from 3 different donor materials. (B) The identification of commonly expressed miRNA between 3 different FBS-EV products manufactured from 3 different donor materials. (C) The identification of the commonly expressed miRNA and the unique miRNA in the PLT and FBS groups. (D) The identification of common and unique miRNA gene targets. EVs: Extracellular vesicles; PLT: human platelet lysate; FBS: fetal bovine serum.

**Figure 4.**
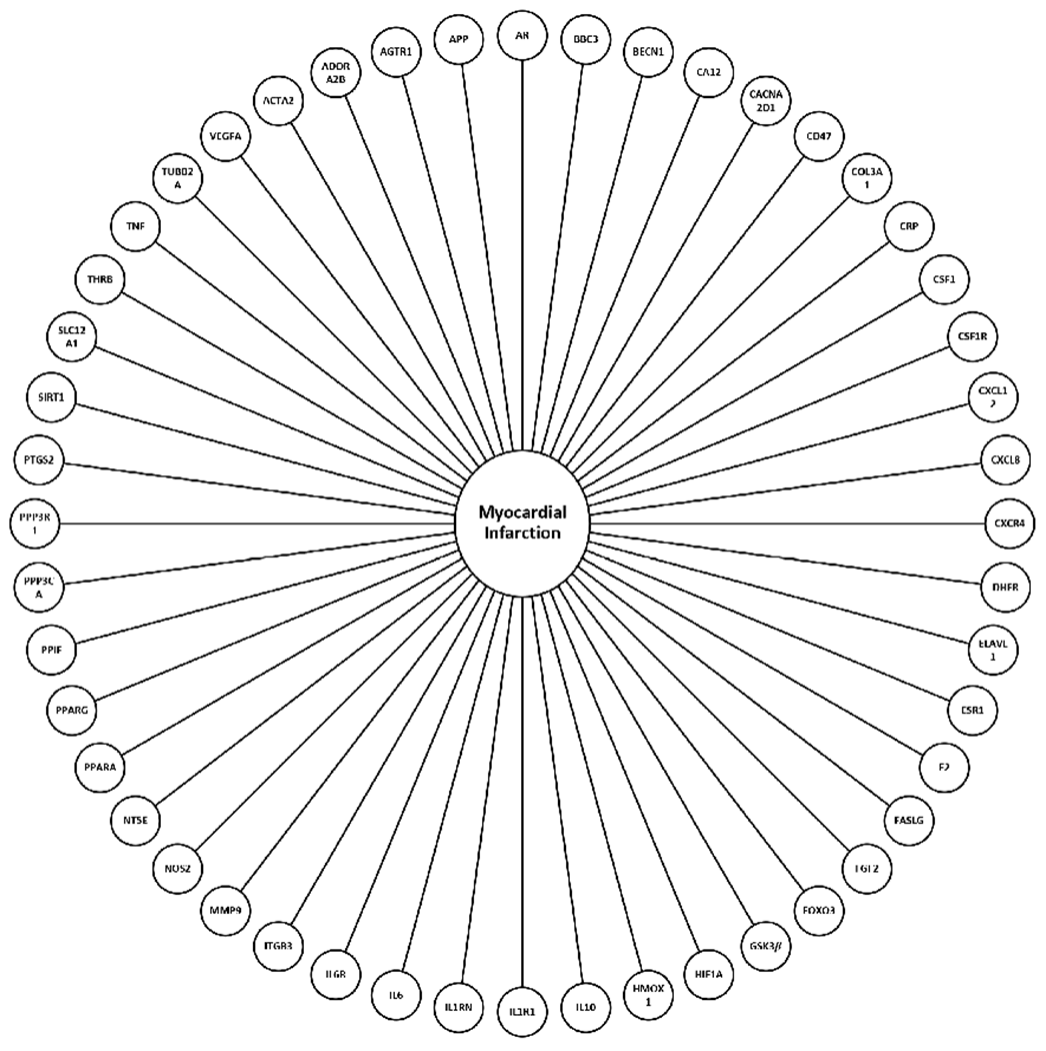
Bioinformatics analysis of MI gene targets. WJMSC EV miRNA gene targets associated with MI. MI: Myocardial infarction; WJMSC: Wharton’s Jelly-derived mesenchymal stem cell; EV: extracellular vesicle.

Sequencing of the miRNA cargo was completed using the three-donor manufactured WJMSC-EVs in triplicate. Results revealed 157 mature miRNAs in PLT-EVs and 154 mature miRNAs in FBS-EVs with greater than 100 copies [Figure 3A and B, Supplementary Table 1]. Direct comparison between the two groups showed 142 commonly expressed miRNAs, 15 unique miRNAs in the PLT group, and 12 unique miRNAs in the FBS group [Figure 3C, Supplementary Table 2]. Qiagen IPA bioinformatics analysis connected 68 miRNAs to 1024 mRNA targets in the PLT group and 64 miRNAs to 1048 mRNA in the FBS group. Comparison of these mRNA targets showed 993 common mRNA targets with 28 unique targets in the PLT group and 52 unique targets in the FBS group [Figure 3D, Supplementary Tables 3 and 4]. Analysis of miRNA targets was completed to identify gene targets associated with MI [Figure 4].

### Evaluation of EV administration post-MI by echocardiography

**Figure 5.**
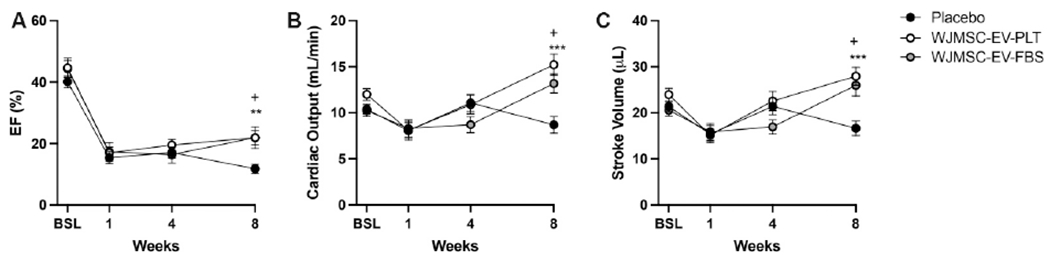
Echocardiography measurements of cardiac function parameters. (A) Ejection fraction percentage at each time point. (B) Cardiac output at each time point. (C) Stroke volume at each time point. (D) Fractional shortening at each time point. Baseline *n* = 10-13 in all groups, week 1, 4, and 8 *n* = 14. **P < 0.01, ***P < 0.001, ^+^*P* < 0.05. EF: Ejection fraction; WJMSC: Wharton’s Jelly-derived mesenchymal stem cell; EV: extracellular vesicle; PLT: human platelet lysate; FBS: fetal bovine serum.

Cardiac function was assessed in adult male mice by echocardiography at baseline and after MI at weeks 1, 4, and 8. One week after myocardial injury, mice were randomized into placebo and EV treatment groups. Re-evaluation of cardiac function at 4-week showed no significant changes in the EV treated group compared to placebo. However, significant improvement in EF was evident at 8-week in both the WJMSC-EV-PLT and WJMSC-EV-FBS groups *vs*. placebo [Figure 5A, Table 1]. Similarly, EV administration significantly elevated CO after 8-week compared to placebo [Figure 5B, Table 1] and significantly rescued SV [Figure 5C, Table 1]. No significant changes in fractional shortening, cardiac volume, or area parameters were observed [Table 1]. Heart rate, body weight, and other cardiac structure parameters were unaffected by the treatment [Table 1].

### Integrated hemodynamic responses to EV treatment post MI

**Figure 6.**
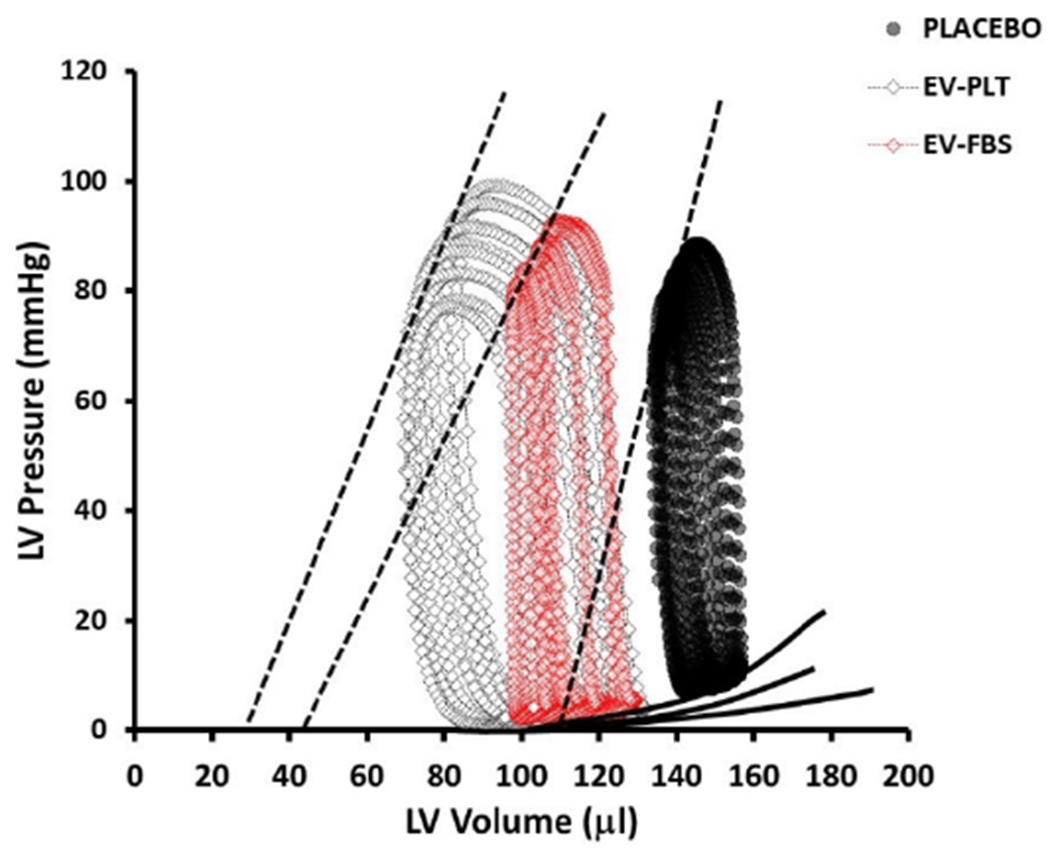
Hemodynamic PV loop measurements. Representative PV loops from each experimental group. EV: Extracellular vesicle; PLT: human platelet lysate; FBS: fetal bovine serum; LV: left ventricle; PV: pressure-volume.

**Figure 7.**
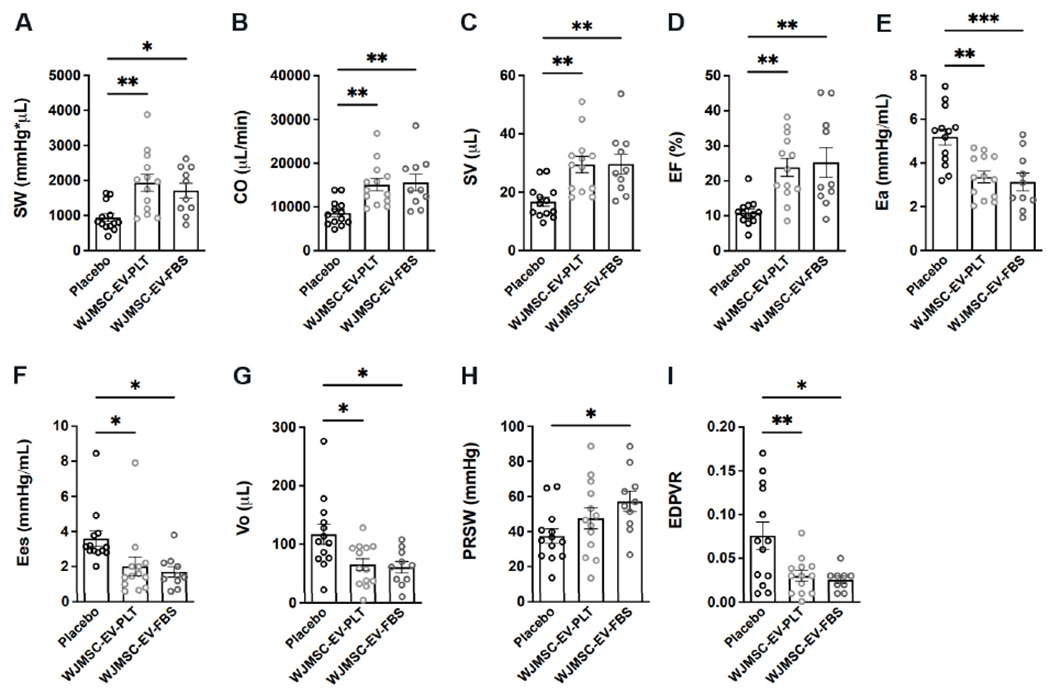
Hemodynamic PV loop measurements of cardiac function parameters. PV loop analysis of: (A) stroke work (SW); (B) cardiac output (CO); (C) stroke volume (SV); (D) ejection fraction (EF); (E) arterial elastance (Ea); (F) slope of end-systolic pressure-volume relationship (Ees); (G) volume-axis intercept of ESPVR (Vo); (H) preload recruitable stroke work (PRSW); and (I) slope of end-diastolic pressure-volume relationship (EDPVR). Placebo *n* = 13, WJMSC-EV-PLT *n* = 13, WJMSC-EV-FBS *n* = 10. *P < 0.05, **P < 0.01, ***P <001. WJMSC: Wharton’s Jelly-derived mesenchymal stem cell; EV: extracellular vesicle; PLT: human platelet lysate; FBS: fetal bovine serum.

Left ventricular micromanometry catheterization was performed 7-week after the first EV administration to assess hemodynamic parameters within each group [Figure 6, Table 2]. Administration of both EV preparations similarly improved cardiac parameters of integrated performance, afterload, contractility, and lusitropy. Integrated performance parameters found to be increased in both EV groups include stroke work [Figure 7A, Table 2], CO [Figure 7B, Table 2], SV [Figure 7C, Table 2], and EF [Figure 7D, Table 2]. At the same time, the arterial-ventricular coupling ratio (Ea/Ees) was not different among groups [Table 2]. Afterload, indexed by arterial elastance (Ea), was significantly reduced in both EV treatment groups [Figure 7E, Table 2]. Although the slope of the end-systolic pressure-volume relationship (Ees) [Figure 7F, Table 2] was lower in the EV treated groups, the volume-axis intercept of ESPVR (Vo) [Figure 7G, Table 2] was substantially shifted leftward, indicating a positive effect on contractility. This was further supported by a marked increase in preload recruitable stroke work in WJMSC-EV-FBS *vs*. placebo [Figure 7H, Table 2]. Lastly, the slope of end-diastolic pressure-volume relationship significantly decreased in both EV groups, consistent with improved chamber compliance [Figure 7I, Table 2].

### Quantification of infarct size

**Figure 8.**
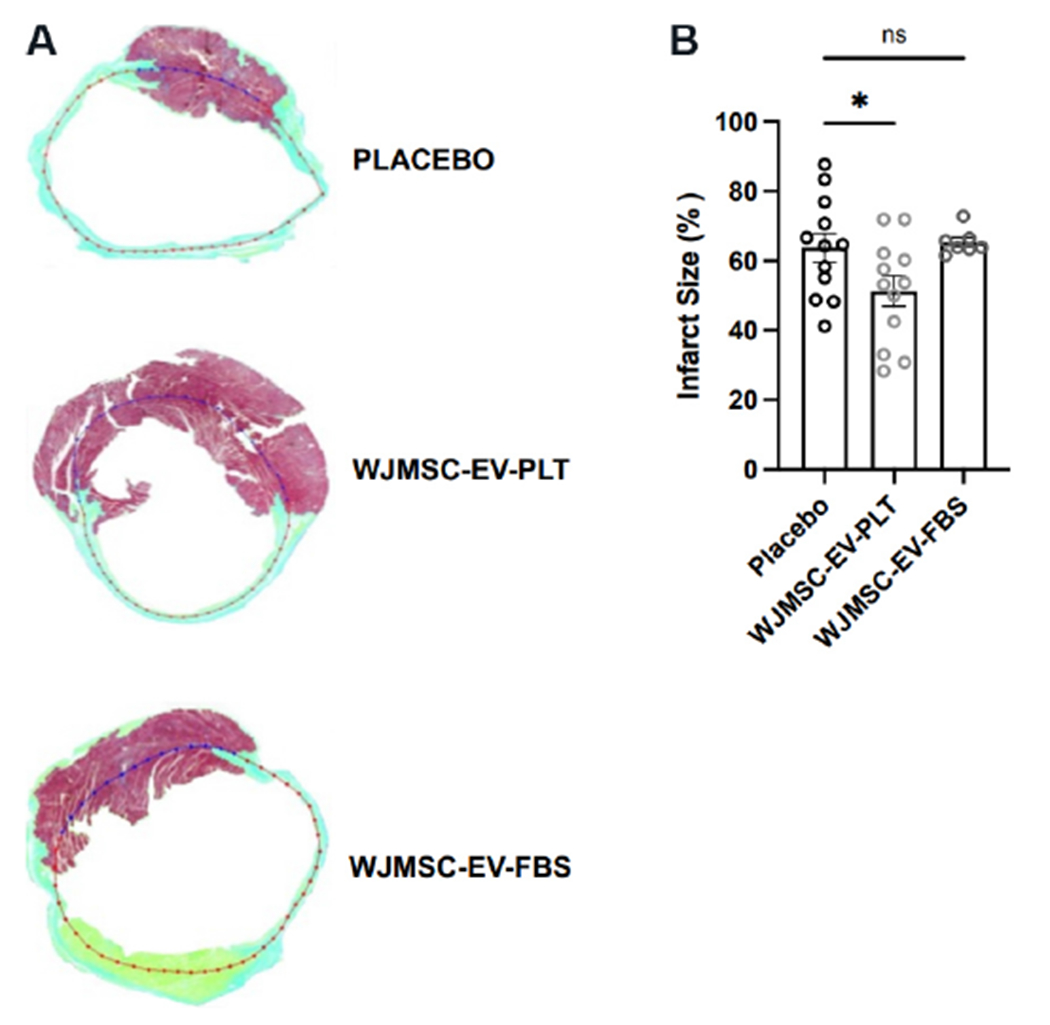
Myocardial infarction size. (A) Representative Masson’s trichrome staining of heart sections at the midventricular level of each treatment group. (B) Comparative quantification of infarct size percentage. Placebo *n* = 12, WJMSC-EV-PLT *n* = 12, WJMSC-EV-FBS *n* = 7. *P < 0.05. WJMSC: Wharton’s Jelly-derived mesenchymal stem cell; EV: extracellular vesicle; PLT: human platelet lysate; FBS: fetal bovine serum.

Masson’s trichrome staining of myocardial histology sections was completed to compare infarct size at the 8-week endpoint [Figure 8A]. The quantification of infarct percentage compared to healthy myocardium allowed for the determination of cardiac injury. Interestingly, the infarct size in each EV group compared to the placebo control showed a significant decrease only in the WJMSC-EV-PLT group (51.3% ± 4.3% in WJMSC-EV-PLT *vs*. 63.9% ± 4.1% in placebo, *P* < 0.05, Figure 8B).

## DISCUSSION

The translation of EV therapy has been challenged by the need for large-scale production of EVs in an efficient and reproducible manner^[15]^. Typical cell culture expansion of MSCs in flasks can produce sufficient EV numbers for small animal experiments. However, for translation of EV doses to humans, EV production would need to expand significantly to produce sufficient numbers of EVs for multiple patients or potentially multiple doses for a single patient. The Quantum bioreactor is established as an effective tool for MSC expansion and has recently been adopted by several cell production faculties to achieve their large-scale production goals^[27]^.

The Quantum bioreactor contains a 3-dimensional hollow-fiber closed bioreactor with approximately 11,500 hollow fibers that provide 2.1 m^2^ of cell growth surface area, which is roughly equivalent to 120 T-flasks (175 mm^2^). Inward and outward media flow lines facilitate the supply of fresh media to cells and the collection of EV-conditioned media. Our study has further demonstrated that the Quantum bioreactor is an effective tool for large-scale WJMSC expansion and subsequent EV isolation. In this study, we demonstrate the superiority of PLT media expansion in the Quantum bioreactor for high cell yields compared to FBS supplemented expansion. Furthermore, those PLT expanded cells tended to produce a slightly greater yield of EV particles compared to the FBS expanded counterpart. Although this result was not significant, the higher cell expansion and reproducibility of EV particle yield suggest PLT as the optimal expansion media choice for EV harvest in serum-free media. Our EV production methodology included sequential centrifugation and precipitation at 100,000 x*g* to concentrate the fraction of small EVs. Nanoparticle analysis and flow cytometry confirmed the presence of small EVs. These products had a particle mode size below 150 nm and were positive for CD63 and CD81^[28]^.

The suitability of the Quantum bioreactor as a large-scale platform for EV production is further demonstrated by the total EV production yield and the production of clinically relevant size doses. Although optimized EV doses have yet to be established and proven in the clinic, small and large animal studies have established ranges of effective doses to be considered for clinical use. Reported doses tested in mice ranged from 1 × 10^9^ to 3 × 10^10^ total particles, whereas pig models have EV doses ranging from 1 × 10^12^ to 3 × 10^12^ total particles per dose^[29,30]^. With the assumption that a human dose would be similar to that of a large animal, our methodology was able to produce a minimum of 3-10 clinical doses of EV from a single 2-week long manufacturing run. This methodology easily meets the need of initial phase I and II clinical trials designed to test the safety and efficacy of MSC-derived EVs.

While it is well established that PLT is an effective alternative to FBS for WJMSC expansion without altering the therapeutic properties, it is unknown if expansion media modulates EV cargo. EV cargo, particularly miRNA, plays a vital role in cardio-protection and cardiac regeneration when transferred to damaged cells^[31,32]^. Therefore, we identified the EV miRNA cargo produced from the FBS and PLT methods. We found few variations in miRNA profiles with regard to miRNA content. Most of the miRNA content was shared between the FBS and PLT products, and bioinformatics analysis found multiple Mi-associated gene targeting in similarly expressed miRNA. The identified uniquely expressed miRNAs all fell under 1075 read counts, with the majority under 400 read counts. Therefore, the uniquely targeted gene pathways may not provide a significant therapeutic advantage.

Following the large-scale manufacturing of the WJMSC EV products, we proceeded to test their therapeutic potential in our established model of MI. We sought to investigate if these products could rescue cardiac function after an established myocardial infarction injury when delivered in a systemic manner. Due to the large EV product yield, we were able to explore this proof-of-concept experiment by delivering multiple EV administrations throughout the study time course. Since the intrajugular injection is an indirect delivery route of EVs to target the injury site, we explored a repeated administration strategy to determine a potential therapeutic effect. We note that additional experiments maybe required to determine the optimal dosing strategy.

We started administering the EV treatment 1 week after the established MI injury via intra-jugular injection. Subsequent injections were completed every week for 6 weeks, and an analysis of the animals was performed at 8 weeks post-MI to determine long-term changes to cardiac function from the repeated administration. It is important to note that no reported adverse side effects were noticed with the multiple injections. With our current treatment strategy, both echocardiography and hemodynamic analysis confirmed EV-mediated improvements in cardiac function. Important parameters such as integrated performance, afterload, contractility, and lusitropy were all found to be significantly improved by the EV treatment. Surprisingly, Ees was reduced in both treated groups; however, this finding occurred in the presence of a substantial leftward shift in Vo indicated an extreme degree of remodeling, a situation where changes in afterload can affect Ees^[33–35]^. In addition, as the ventricular pressure gets down to ~50-60 mmHg, the heart perfusion is limited, resulting in a curvilinear instead of linear response^[36,37]^. Changes in Ees can also reflect changes in cardiac morphometry such as hypertrophy and fibrosis; thus, it can misrepresent contractile function^[38,39]^. On the other hand, Vo provides a good LV contractility index due to less sensitivity to large changes in afterload conditions^[35]^. Accordingly, a higher Vo in the placebo group reflects lower contractility.

Aside from function, a decrease in cardiac infarct size was observed in the WJMSC-EV-PLT group, indicating a possible anti-fibrotic effect that may have contributed to an improvement in cardiac function. Surprisingly, this anti-fibrotic effect was not observed in the WJMSC-EV-FBS group. We hypothesize that the lack of improvement in cardiac fibrosis with the FBS-EVs may be related to limited retention of EVs in the myocardium compared to the PLT-EVs, which may have led to differences in the reduction in the infarction microenvironment. Instead, both FBS-EV and PLT-EV products improved cardiac function, perhaps due to a systemic effect that was achieved through the modulation of remote myocardial remodeling. The coronary microvasculature undergoes increased vascular permeability and endothelial dysfunction associated with MI^[40]^. Accordingly, effective cardioprotective effects include not only infarct size reduction but also positive effects on coronary microcirculation, inflammation, and vascular integrity^[41]^. Therefore, future studies to define the contribution of coronary microcirculation and extend the time-frame follow-up of cardiac performance are warranted to elucidate the therapeutic efficacy of EVs. Nonetheless, the improvements in cardiac function along with reduced infarction size with the PLT-EV treatment make this product an important candidate for therapeutic trials.

Previous publications have suggested several mechanisms of action for EV-induced cardiac regeneration^[42]^, including the transfer of miRNA EV cargo to restore signaling pathways that promote angiogenesis, macrophage polarization, and decreased fibrosis^[43–46]^. With the vast targeting of miRNAs, it is likely that EV-induced repair is not limited to a single mechanism but is, in fact, a coordinated effect induced by multiple pathways.

In conclusion, our work demonstrates the feasibility of using large-scale expansion systems to produce high quantities of potent EVs for regenerative therapies. This study serves as a starting point for EV translation into clinical research via a validated manufacturing procedure and demonstrated proof-of-concept animal study. Furthermore, these results have potential clinical implications for the treatment of MI and other cardiac injuries.

## Supplementary Material

Supplementary Materials

## Figures and Tables

**Table 1.** Echocardiography measurements of cardiac function parameters

	Time point	Placebo	WJMSC-EV-PLT	WJMSC-EV-FBS
Ejection fraction (%)	Baseline	40.1 ± 1.9	44.7 ± 3.2	44.3 ± 2.7
	Week 1	15.4 ± 1.9	16.9 ± 1.9	17.3 ± 3.0
	Week 4	17.1 ± 1.8	19.5 ± 1.7	16.4 ± 2.8
	Week 8	11.8 ± 1.4	21.9 ± 2.3**”	21.9 ± 3.5*
Cardiac output (μL)	Baseline	10.4 ± 0.5	12.0 ± 0.6	10.2 ± 0.6
	Week 1	8.06 ± 1.0	8.09 ± 0.8	8.30 ± 0.9
	Week 4	11.1 ± 0.9	10.9 ± 1.0	8.70 ± 0.9
	Week 8	8.70 ± 0.9	15.2 ± 1.2***”	13.2 ± 1.0*
Stroke volume (μL)	Baseline	21.4 ± 1.1	23.9 ± 1.4	20.6 ± 1.3
	Week 1	15.4 ± 1.9	15.1 ± 1.3	15.8 ± 1.8
	Week 4	21.4 ± 1.8	22.5 ± 2.1	16.9 ± 1.5
	Week 8	16.7 ± 1.6	27.9 ± 2.0***	26.0 ± 2.3*
Fractional shortening (%)	Baseline	7.74 ± 0.6	8.82 ± 0.7	7.06 ± 0.6
	Week 1	4.55 ± 0.6	3.15 ± 0.3	4.61 ± 0.6
	Week 4	5.12 ± 0.8	3.98 ± 0.5	4.01 ± 0.6
	Week 8	2.67 ± 0.6	4.21 ± 0.5	4.59 ± 0.5
Volume (μL)	Baseline	51.5 ± 2.5	51.2 ± 3.5	42.7 ± 3.0
	Week 1	105.7 ± 8.6	94.6 ± 9.4	94.0 ± 7.8
	Week 4	122.5 ± 9.6	115.0 ± 13.6	109.7 ± 10.1
	Week 8	140.0 ± 15.2	131.5 ± 14.5	120.0 ± 13.8
Volume-diastolic (μL)	Baseline	53.3 ± 1.2	55.3 ± 3.0	46.7 ± 1.5
	Week 1	110.4 ± 8.5	95.4 ± 8.6	99.2 ± 7.4
	Week 4	135.0 ± 10.8	124.3 ± 14.9	115.7 ± 9.1
	Week 8	156.6 ± 15.4	142.7 ± 14.6	132.5 ± 11.7
Volume-systolic (μL)	Baseline	31.9 ± 1.3	31.3 ± 3.0	26.1 ± 1.7
	Week 1	95.0 ± 8.0	80.2 ± 8.4	83.4 ± 8.0
	Week 4	113.5 ± 10.5	101.8 ± 13.9	98.8 ± 10.0
	Week 8	140.0 ± 15.2	114.7 ± 14.8	106.5 ± 12.7
Area (mm^2^)	Baseline	20.4 ± 0.7	20.4 ± 0.8	18.2 ± 0.8
	Week 1	30.9 ± 1.6	28.6 ± 1.7	28.9 ± 1.4
	Week 4	33.7 ± 1.6	32.2 ± 2.2	31.5 ± 1.7
	Week 8	37.5 ± 2.3	35.4 ± 2.3	32.9 ± 2.3
Area-diastolic (mm^2^)	Baseline	21.0 ± 0.3	21.5 ± 0.7	19.3 ± 0.3
	Week 1	31.8 ± 1.5	29.0 ± 1.6	29.9 ± 1.4
	Week 4	35.9 ± 1.6	33.7 ± 2.2	32.6 ± 1.5
	Week 8	39.0 ± 2.3	37.1 ± 2.2	35.4 ± 1.8
Area-systolic (mm^2^)	Baseline	15.5 ± 0.4	15.3 ± 0.9	13.8 ± 0.4
	Week 1	28.5 ± 1.4	26.0 ± 1.6	26.6 ± 1.6
	Week 4	31.7 ± 1.7	29.6 ± 2.3	29.3 ± 1.8
	Week 8	36.3 ± 2.3	32.2 ± 2.5	30.6 ± 2.2
Heart rate (bpm)	Baseline	487.2 ± 9.6	503.9 ± 12.8	498.3 ± 12.4
	Week 1	525.8 ± 16.7	532.5 ± 25.4	526.2 ± 13.0
	Week 4	518.4 ± 10.0	484.9 ± 11.2	512.2 ± 15.4
	Week 8	518.3 ± 13.3	543.8 ± 13.8	513.5 ± 15.9
Body weight (g)	Baseline	25.9 ± 0.5	24.9 ± 0.4	24.3 ± 0.6
	Week 1	25.5 ± 0.5	24..3 ± 0.6	24.4 ± 0.6
	Week 4	28.6 ± 0.4	27.8 ± 0.4	26.8 ± 0.6
	Week 8	30.5 ± 0.5	29.5 ± 0.4	29.6 ± 0.6

* *P* < 0.05

** *P* < 0.01

*** *P* < 0.001.

WJMSC-EV-PLT: Wharton’s Jelly-derived mesenchymal stem cell-extracellular vesicle-human platelet lysate; WJMSC-EV-FBS: Wharton’s Jelly-derived mesenchymal stem cell-extracellular vesicle-fetal bovine serum.

**Table 2.** Hemodynamic PV loop measurements of cardiac function parameters

	PV parameter	Placebo	WJMSC-EV-PLT	WJMSC-EV-FBS
Integrated performance	SW (mmHg* μL)	940.8 ± 108.8	1935 ± 243.3**	1712 ± 213.6*
	CO (μL/min)	8619 ± 811.0	15079 ± 1391**	15578 ± 1909**
	SV (μL)	16.7 ± 1.6	29.5 ± 2.8**	29.6 ± 3.5**
	EF (%)	10.9 ± 1.0	23.85 ± 2.6**	25.29 ± 4.3**
	Ea/Ees	1.77 ± 0.2	2.67 ± 0.4	2.36 ± 0.6
Afterload	ESP (mmHg)	81.1 ± 3.9	91.9 ± 4.9	81.6 ± 4.5
	Ea (mmHg/mL)	5.20 ± 0.4	3.36 ± 0.3**	3.13 ± 0.4***
Preload	EDP (mmHg)	13.5 ± 1.6	14.9 ± 1.8	14.6 ± 2.3
	EDV (μL)	163.7 ± 15.7	132.3 ± 9.6	133.3 ± 11.2
Contractility	dP/dt max (mmHg/s)	5005 ± 263.0	5637 ± 348.0	5375 ± 392.6
	Ees (mmHg/mL)	3.6 ± 0.4	2.0 ± 0.5*	1.7 ± 0.3*
	PRSW (mmHg)	37.4 ± 4.2	47.6 ± 5.9	57.2 ± 5.7*
	dP/dt max_EDV	108.4 ± 12.2	99.4 ± 21.4	108.4 ± 22.4
	Vo (μL)	116.8 ± 17.4	65.1 ± 10.0*	60.5 ± 9.7*
Lusitropy	dP/dt min (mmHg/s)	−4113 ± 258	−4664 ± 345	−4472 ± 431
	Tau (ms)	11.8 ± 0.8	11.4 ± 0.7	11.7 ± 0.9
	EDPVR	0.07 ± 0.02	0.03 ± 0.006**	0.03 ± 0.004*
	ESV (μL)	142.7 ± 16.6	104.9 ± 12.0	107.4 ± 12.8
	Heart rate (bpm)	514.0 ± 4.5	517.2 ± 11.6	523.9 ± 6.2

* *P* < 0.05

** *P* < 0.01

*** *P* < 0.001.

PV: Pressure-volume; WJMSC-EV-PLT: Wharton’s Jelly-derived mesenchymal stem cell-extracellular vesicle-human platelet lysate; WJMSC-EV-FBS: Wharton’s Jelly-derived mesenchymal stem cell-extracellular vesicle-fetal bovine serum; SW: stroke work; CO: cardiac output; SV: stroke volume; EF: ejection fraction; Ea/Ees: arterial-ventricular coupling ratio; ESP: end-systolic pressure; Ea: arterial elastance; EDP: end-diastolic pressure; EDV: end-diastolic volume; Ees: slope of end-systolic pressure-volume relationship; PRSW: preload recruitable stroke work; Vo: volume-axis intercept of ESPVR; EDPVR: end-diastolic pressure-volume relationship; ESV: end-systolic volume.
