# Supplementary material for: Systemic delivery of large-scale manufactured Wharton’s Jelly mesenchymal stem cell-derived extracellular vesicles improves cardiac function after myocardial infarction
Source: J Cardiovasc Aging. Author manuscript; Available in PMC 2022 Feb 1. (PMC8804674; doi:10.20517/jca.2021.21)
Supplement: Supplementary Materials [file NIHMS1770304-supplement-Supplementary_Materials.pdf]

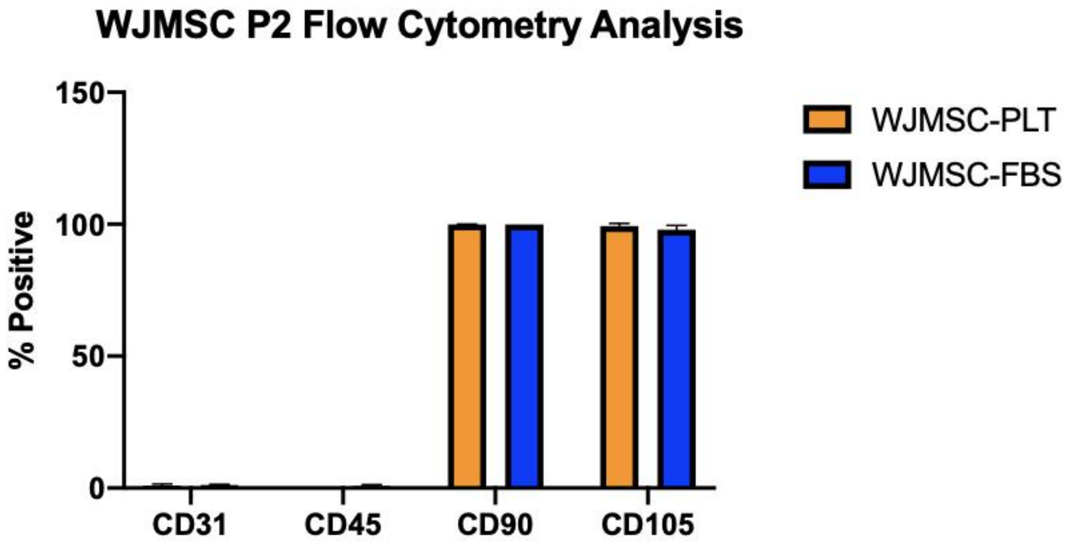

Supplementary Figure 1. WJMSC characterization. Flow cytometry analysis of WJMSC at passage 2 confirms the positive expression of CD90 and CD105 and the negative expression of CD31 and CD45. WJMSC: Wharton's Jelly-derived mesenchymal stem cell; PLT: human platelet lysate; FBS: fetal bovine serum.

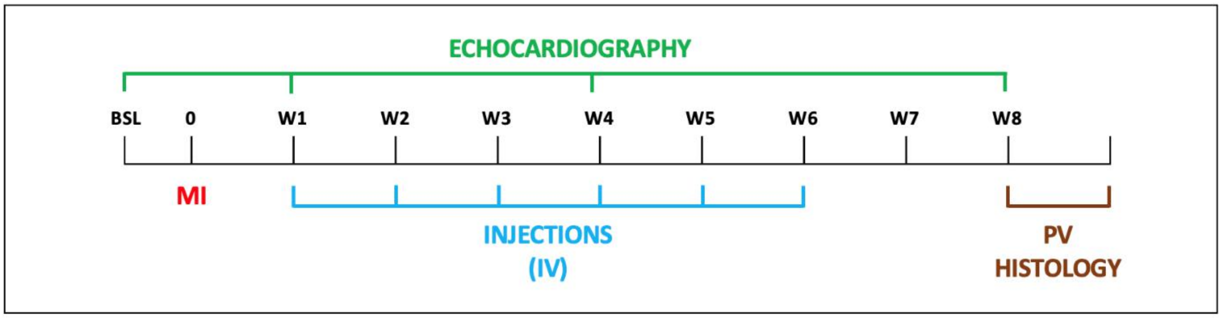

Supplementary Figure 2. Experimental timeline. The experimental timeline outlining the 8 weeks study period. 1 week after MI, EV product was administered IV 1 time a week for 6 weeks. Echo was completed at BSL, week 1, week 4, and week 8. PV loop and histology were completed at week 8. MI: Myocardial infarction; EV: extracellular vesicle.

Supplementary Table 1. miRNA sequencing read counts

PLT-MSC EVs miRNA		FBS-MSC EVs miRNA	
**Gene name**	**Average reads**	**Gene name**	**Average reads**
hsa-mir-21	202389.7518	***hsa-mir-21***	158216.3446
hsa-let-7i	69472.48133	***hsa-let-7i***	89320.84331
hsa-mir-29a	67673.4987	***hsa-mir-143***	73626.70747
hsa-mir-143	61172.24088	***hsa-mir-29a***	63852.83203
hsa-mir-221	56561.60069	***hsa-let-7b***	57850.62717
hsa-mir-7641-2	48872.94607	***hsa-mir-146a***	38678.20856
hsa-mir-146a	44752.59332	***hsa-mir-221***	34352.31489
hsa-let-7b	31252.79406	***hsa-let-7e***	29311.72114
hsa-mir-34a	23614.31574	***hsa-mir-125a***	24353.86806
hsa-mir-148a	22504.8444	***hsa-mir-148a***	21364.98916
hsa-mir-31	18844.45639	***hsa-mir-7641-2***	17675.02907
hsa-let-7e	14091.18392	***hsa-mir-432***	15963.76606
hsa-mir-100	13718.98059	***hsa-mir-31***	15519.82596
hsa-mir-125a	13038.06673	***hsa-mir-382***	15143.48741
hsa-mir-23a	12389.41896	***hsa-mir-423***	14856.98336
hsa-mir-26b	10012.92436	***hsa-mir-100***	14401.15169
hsa-mir-155	9649.584589	***hsa-mir-23a***	13075.9986
hsa-mir-199b	9225.6143	***hsa-mir-191***	11966.22352
hsa-mir-191	8415.623922	***hsa-mir-320a***	11142.41883
hsa-mir-127	8230.132811	***hsa-mir-151a***	8843.3987
hsa-mir-27b	6838.826422	***hsa-mir-127***	8800.054678
hsa-mir-27a	6686.583678	***hsa-mir-409***	8783.1678
hsa-mir-409	6616.611011	***hsa-mir-155***	8389.892111
hsa-mir-503	6511.589522	***hsa-mir-27b***	8371.643522
hsa-mir-10a	6075.206989	***hsa-mir-99b***	7442.533356
hsa-mir-93	6002.5415	***hsa-mir-196b***	7321.798678
hsa-mir-151a	5770.942289	***hsa-mir-26b***	6103.3089

hsa-mir-432	5593.335011	***hsa-mir-30a***	6029.036478
hsa-let-7g	5530.109289	***hsa-mir-30d***	5995.4942
hsa-mir-222	5337.405567	***hsa-mir-93***	5929.749956
hsa-mir-424	5289.317511	***hsa-mir-27a***	5656.706067
hsa-mir-25	5185.945856	***hsa-mir-34a***	5588.682444
hsa-let-7c	5090.951467	***hsa-mir-25***	5321.087567
hsa-mir-382	5033.314133	***hsa-mir-10a***	5318.612633
hsa-mir-196b	4991.435	***hsa-mir-222***	5256.918233
hsa-mir-654	4930.650933	***hsa-mir-134***	5032.463556
hsa-mir-30d	4565.347578	***hsa-mir-654***	5005.345722
hsa-mir-493	4552.016611	***hsa-mir-196a-2***	4896.8769
hsa-mir-99b	4482.341033	***hsa-mir-342***	4733.303344
hsa-mir-320a	4452.056733	***hsa-mir-125b-1***	4614.000633
hsa-mir-30a	4208.714022	***hsa-let-7g***	4440.585711
hsa-mir-152	4179.804467	***hsa-mir-381***	4038.711356
hsa-mir-708	4105.965733	***hsa-mir-493***	4004.042122
hsa-mir-423	4091.460422	***hsa-mir-214***	3810.103511
hsa-mir-148b	4038.811878	***hsa-mir-379***	3752.624433
hsa-mir-134	3869.791911	***hsa-mir-574***	3655.895989
hsa-mir-196a-2	3707.052756	***hsa-mir-224***	3635.529433
hsa-mir-22	3450.782722	***hsa-mir-148b***	3300.377856
hsa-mir-342	3400.091844	***hsa-mir-708***	3214.915933
hsa-mir-379	3316.400222	***hsa-mir-145***	3157.132822
hsa-mir-214	2965.195722	***hsa-mir-132***	3060.151578
hsa-mir-574	2948.291633	***hsa-let-7f-2***	2797.853956
hsa-mir-381	2931.172444	***hsa-mir-152***	2749.6317
hsa-mir-145	2775.811267	***hsa-mir-370***	2744.505856
hsa-mir-140	2684.237311	***hsa-mir-503***	2644.096844
hsa-mir-30e	2677.400733	***hsa-let-7c***	2546.679567
hsa-mir-335	2638.985589	***hsa-mir-369***	2467.8796

hsa-mir-370	2517.349111	***hsa-mir-22***	2234.536789
hsa-mir-10b	2418.155367	***hsa-mir-361***	2203.599822
hsa-mir-125b-1	2278.842644	***hsa-mir-431***	2072.700433
hsa-let-7f-2	2159.046767	***hsa-mir-30e***	2015.313033
hsa-mir-376c	2099.947989	***hsa-mir-140***	1972.377578
hsa-mir-28	2039.237078	***hsa-mir-335***	1927.848567
hsa-mir-425	1986.233556	***hsa-mir-193a***	1865.593311
hsa-mir-361	1898.102267	***hsa-mir-425***	1800.810567
hsa-mir-369	1884.606211	***hsa-mir-1246***	1767.811622
hsa-mir-411	1735.109956	***hsa-mir-424***	1718.147511
hsa-mir-431	1725.044267	***hsa-mir-10b***	1713.916733
hsa-mir-136	1703.801867	***hsa-mir-23b***	1704.330089
hsa-mir-132	1664.275522	***hsa-let-7d***	1615.237689
hsa-let-7d	1525.696589	***hsa-mir-411***	1578.556778
hsa-mir-224	1514.768844	***hsa-mir-28***	1567.024633
hsa-mir-337	1498.9928	***hsa-mir-337***	1312.842556
hsa-mir-23b	1445.808711	***hsa-mir-98***	1307.151178
hsa-mir-186	1434.926967	***hsa-mir-199b***	1306.301378
hsa-mir-137	1387.040733	***hsa-mir-1307***	1216.227644
hsa-mir-15b	1355.277867	***hsa-mir-186***	1173.139867
hsa-mir-130a	1139.7994	***hsa-mir-323a***	1171.379244
hsa-mir-494	1139.717333	***hsa-mir-142***	1115.633644
hsa-mir-146b	1108.602578	***hsa-mir-137***	1098.538722
hsa-mir-20a	1078.098356	***hsa-mir-15b***	1092.301944
hsa-mir-98	1066.804478	***hsa-mir-122***	1074.585033
hsa-mir-339	1066.636056	***hsa-mir-485***	1044.525611
hsa-mir-487b	1031.674144	***hsa-mir-6087***	1029.943533
hsa-mir-671	1029.595289	***hsa-mir-671***	1028.268811
hsa-mir-455	962.6182222	***hsa-mir-136***	1016.014078
hsa-mir-665	950.2608333	***hsa-mir-376c***	999.4390222

hsa-mir-542	918.9008778	***hsa-mir-487b***	959.9895333
hsa-mir-454	856.4460778	***hsa-mir-199a-2***	955.1862667
hsa-mir-1307	837.8247444	***hsa-mir-494***	942.6891667
hsa-mir-106b	804.4620556	***hsa-mir-16-1***	937.8173333
hsa-mir-7704	763.1122111	***hsa-mir-20a***	878.2891556
hsa-mir-6087	743.5692778	***hsa-mir-3929***	825.0446667
hsa-mir-199a-2	725.7732889	***hsa-mir-130a***	785.8688556
hsa-mir-193a	712.0917889	***hsa-mir-339***	778.7449333
hsa-mir-4532	705.3736333	***hsa-mir-181a-2***	776.0269556
hsa-mir-374a	692.5919778	***hsa-mir-5096***	773.6895667
hsa-mir-323a	688.4405889	***hsa-mir-4516***	744.3336889
hsa-mir-99a	677.9878	***hsa-mir-744***	737.989
hsa-mir-142	676.8422556	***hsa-mir-106b***	692.1451111
hsa-mir-29c	669.2403444	***hsa-mir-17***	690.3638556
hsa-mir-1273a	668.5974778	***hsa-mir-126***	685.0994556
hsa-mir-17	648.8254444	***hsa-mir-629***	662.7819222
hsa-mir-532	618.7978444	***hsa-mir-185***	653.0764667
hsa-mir-185	618.7239222	***hsa-mir-665***	631.0107
hsa-mir-181a-2	598.9191667	***hsa-mir-454***	618.3349222
hsa-mir-154	590.4103	***hsa-mir-154***	560.4866222
hsa-mir-126	543.7228444	***hsa-mir-542***	530.9536556
hsa-mir-130b	524.7776333	***hsa-mir-615***	508.3944778
hsa-mir-615	494.0281222	***hsa-mir-92b***	499.9266556
hsa-mir-1246	481.3837444	***hsa-mir-146b***	492.4965
hsa-mir-485	478.2787556	***hsa-mir-532***	479.5470333
hsa-mir-660	476.6780556	***hsa-mir-455***	477.4843778
hsa-mir-16-1	435.6969222	***hsa-mir-29c***	449.4682
hsa-mir-5096	417.6017222	***hsa-mir-130b***	446.0732111
hsa-mir-299	414.7932556	***hsa-mir-328***	430.6737222
hsa-mir-450b	413.8984111	***hsa-mir-323b***	416.9108778

hsa-mir-345	413.4423889	***hsa-mir-484***	406.4600889
hsa-mir-629	412.1395222	***hsa-let-7f-1***	405.8193667
hsa-mir-484	406.7610667	***hsa-mir-4532***	396.0914333
hsa-mir-30b	402.3682444	***hsa-mir-374a***	389.6246222
hsa-mir-3929	395.2320556	***hsa-mir-345***	370.1651667
hsa-mir-324	392.9544222	***hsa-mir-125b-2***	364.9741333
hsa-mir-190a	356.2202111	***hsa-let-7a-3***	361.1676556
hsa-mir-15a	348.9244778	***hsa-mir-889***	356.5265667
hsa-mir-34c	348.8685778	***hsa-mir-660***	356.4528333
hsa-mir-376a-2	345.9914667	***hsa-mir-92a-1***	337.3121222
hsa-mir-210	345.3609556	***hsa-mir-197***	332.0180222
hsa-mir-92a-1	339.8735778	***hsa-mir-16-2***	328.8195222
hsa-mir-377	328.8209889	***hsa-mir-324***	327.9566778
hsa-let-7f-1	326.5786	***hsa-mir-625***	320.2548111
hsa-mir-889	318.4822889	***hsa-mir-181a-1***	310.7142
hsa-mir-197	302.9086444	***hsa-mir-192***	302.8046444
hsa-mir-378a	301.3257111	***hsa-mir-495***	299.6352556
hsa-mir-92b	300.0858111	***hsa-let-7a-1***	297.0850778
hsa-mir-769	272.0033556	***hsa-mir-296***	277.9803111
hsa-mir-181a-1	269.7242778	***hsa-let-7a-2***	277.8541778
hsa-mir-19a	251.5362	***hsa-mir-450b***	273.9452556
hsa-mir-125b-2	250.8823778	***hsa-mir-15a***	268.7519222
hsa-mir-625	246.4861333	***hsa-mir-190a***	267.1790556
hsa-mir-744	245.9201444	***hsa-mir-299***	263.351
hsa-mir-135b	245.1019111	***hsa-mir-3615***	260.5662
hsa-mir-192	243.7499444	***hsa-mir-769***	231.3506889
hsa-mir-495	235.1820444	***hsa-mir-1180***	214.5451111
hsa-mir-181b-2	229.8289333	***hsa-mir-452***	212.3563667
hsa-mir-675	229.3182222	***hsa-mir-196a-1***	207.2698778
hsa-mir-323b	225.4393778	***hsa-mir-576***	194.4725333

hsa-mir-128-1	221.7729778	***hsa-mir-128-1***	187.0343222
hsa-mir-4516	205.2658889	***hsa-mir-181b-2***	185.6746111
hsa-mir-18a	182.3394778	***hsa-mir-34c***	185.3921
hsa-mir-16-2	180.4811222	***hsa-mir-377***	185.3741778
hsa-mir-1271	178.584	***hsa-mir-664a***	164.3350778
hsa-mir-590	176.5045	***hsa-mir-30b***	159.8810556
hsa-mir-1287	154.8084556	***hsa-mir-376a-2***	117.1762111
hsa-mir-376b	147.9224444		
hsa-mir-32	145.6265		
hsa-mir-421	145.1037333		

PLT-MSC: human platelet lysate-mesenchymal stem cell; FBS-MSC: fetal bovine serum-mesenchymal stem cell; EVs: extracellular vesicles.

Supplementary Table 2. Unique miRNA counts

**PLT-MSC EVs**		**FBS-MSC EVs**	
**Gene name**	**Average counts**	**Gene name**	**Average counts**
hsa-mir-7704	763.1122111	***hsa-mir-122***	1074.585033
hsa-mir-99a	677.9878	***hsa-mir-328***	430.6737222
hsa-mir-1273a	668.5974778	***hsa-let-7a-3***	361.1676556
hsa-mir-210	345.3609556	***hsa-let-7a-1***	297.0850778
hsa-mir-378a	301.3257111	***hsa-mir-296***	277.9803111
hsa-mir-19a	251.5362	***hsa-let-7a-2***	277.8541778
hsa-mir-135b	245.1019111	***hsa-mir-3615***	260.5662
hsa-mir-675	229.3182222	***hsa-mir-1180***	214.5451111
hsa-mir-18a	182.3394778	***hsa-mir-452***	212.3563667
hsa-mir-1271	178.584	***hsa-mir-196a-1***	207.2698778
hsa-mir-590	176.5045	***hsa-mir-576***	194.4725333
hsa-mir-1287	154.8084556	***hsa-mir-664a***	164.3350778
hsa-mir-376b	147.9224444		
hsa-mir-32	145.6265		

hsa-mir-421	145.1037333		

PLT-MSC: human platelet lysate-mesenchymal stem cell; FBS-MSC: fetal bovine serum-mesenchymal stem cell; EVs: extracellular vesicles.

Supplement Table 3. Complete list of common mRNA targets from miRNA bioinformatic analysis

AARSD1	EIF3J	NXN	THBS1
ACP1	EIF4G2	OTULINL	TLR4
ADGRG1	F2	PGRMC1	TPM2
AGO4	FADS2	POLD2	TRIM71
AKAP8	FANCD2	POLR2C	TRMT1
ANAPC1	FNDC3A	POM121/POM121C	TTC9C
ATAD3B	GAK	PPP1R7	TUSC2
ATP6V0A1	GEMIN7	PRDM1	TYMS
ATP6V1F	GRPEL2	PRIM1	UGT8
AURKB	GTPBP3	PRRC2A	UHRF1
BCL2L1	GYS1	PTGS2	VIM
BCL7A	HMGA1	PXDN	VPS39
BMP2K	HMGA2	RABGAP1L	WNT1
BSG	HMOX1	RAS	FGF16
CALCOCO2	HYOU1	RBM19	FGFR3
CAPG	IFIT5	RDH10	IGF1R
CARHSP1	IFRD1	RHOB	MTOR
CASP3	IGF2BP1	RHOG	PLK1
CCND1	IGF2BP2	RPP38	RPTOR
CDC25A	IGF2BP3	RRP8	SMARCA5
CDIPT	IPO4	RTCA	APAF1
CDK6	ITGB3	SCYL1	HOXA1
CDKAL1	KCNJ16	SEPTIN3	HOXD10
CEMIP2	KLK10	SIGMAR1	KLF4

CHMP2A	KRAS	SLC1A4	NF1
CIAO2A	KRT19	SLC25A1	USF2
COIL	LIN28A	SLC25A13	AP2M1
COL1A2	MARS2	SLC25A24	API5
COMMD9	MED28	SLC25A32	BAK1
CSDE1	MLLT1	SLC38A1	BAX
CSNK1D	MRM1	SMC1A	BMF
DAD1	MRPS24	SMOX	KLK1
DHX57	MRPS33	SNAP23	TP53
DICER1	MTPN	SPCS3	ABTB1
DOCK5	MTRR	SPRYD4	ACSS1
DRD3	MYC	SYPL1	ADAMTS1
DSP	NEDD4	TAF9B	AJUBA
DUSP12	NF2	TAGLN	ALOX5
DUSP23	NRAS	TGFBR1	ANAPC16
APLN	MAP2K7	CSF1	C11orf58
ARID3A	MAZ	HOXA5	CCDC25
ARID3B	MYD88	MAFB	CCNA2
ATP6AP1L	OSBPL9	MEOX2	CDK4
B3GALT4	PCDHB10	SMAD4	CES1
BMPR1B	PCTP	TAC1	CLINT1
CASP6	PERP	ZFPM2	DDR1
CASP7	PIGR	ARHGAP32	DFFA
CBFB	PPT2	CAPN8	DTD1
CBLN2	RABL6	MECP2	EIF4E
CBX7	RBM8A	MMP9	EIF4EBP2
CCR5	RHEBL1	PGC	F11R
CDH5	SCD	RB1	FBXO28
CDKN2A	SGPL1	SOX4	FLI1
CEBPG	SMO	TJP1	FSCN1

CYP1A1	ST18	ALOX5AP	KLF5
DDX19B	TAFA1	HTR1A	KRT7
DIO3	TENM2	RUNX2	LAMP2
DUS1L	TOR2A	SMAD5	MDFI
E2F3	TSPAN8	TRPS1	MMP1
ELAVL1	UBE2I	NCOA2	MUC1
ENTPD4	UGT2B15	ADAMTS5	NDUFA4
ERBB2	UGT2B17	CXCL12	PADI1
ERBB3	UGT2B28	DNPEP	PARP8
GPR160	UVRAG	EGR2	PPP3CA
GSS	VSIR	HDAC4	RASA1
H3-3A/H3-3B	ZNF385A	IGFBP5	ROBO4
H4C1	CRKL	SMAD3	RTKN
HK2	IRS1	BCL2	SPTB
ID1	PIK3R2	DNMT3A	SWAP70
ID2	SLC45A3	FNDC3B	TPM3
ID3	SPRED1	MAPK12	UNG
IGFBP3	TOM1	MAPK7	USP46
IKZF4	VCAM1	MDM2	VASN
IL1RN	VEGFA	PRC1	ATOH8
JARID2	BCL6	TOP2A	BLMH
KLF13	RTL1	ABRACL	BRCA1
LIPA	XBP1	ACBD3	C8A
MAN1A1	ATG2B	AHNAK	CAMP
CCL8	MR1	AICDA	IKBKE
CCR3	NFIX	AMIGO2	IL13RA1
CD1D	NLGN1	ANKFY1	INPP5D
CD40	NOS2	ARFIP1	LCLAT1
CDKN3	NOVA1	ARFIP2	LDOC1
CFH	PA2G4	ARID2	LPL

CHUK	PBLD	ARL10	LY6K
COL13A1	PDGFRA	ARL5B	MAF
CRP	PDIK1L	ATG3	1-Mar
CXCL8	PEX11G	ATP6V1C1	MATR3
CXCR4	PGLYRP1	BACH1	MEIS1
DMBT1	PGLYRP2	BET1	MET
FADD	PLEKHA4	BRPF3	MOSPD2
IFNA1/IFNA13	POLE2	CCN1	MPZL1
IFNB1	PRR15	CD47	MSI2
IL10	PTAFR	CDK5RAP3	MYB
IL12RB2	PTGES2	CEBPB	MYO10
IL1F10	RAD54L	CHAF1A	MYO1E
IL1R1	S100A12	CLDN1	NARS1
IL1RAP	SDCBP2	CSF1R	NT5E
IL1RAPL2	SFTPD	CTLA4	PDE3A
IL1RL2	STAT1	CTNNB1	PDLIM5
IL36A	SYT1	CUL4B	PHC2
IL36B	TIMELESS	CUX1	PICALM
IL36G	TLR1	CYP51A1	PKN2
IL36RN	TLR10	DCAF7	PLXND1
IL37	TLR9	DHX40	PMAIP1
IRAK1	TMSB15A	DNAJB1	PODXL
IRAK2	TRAF6	DNAJC19	POLE3
IRF5	TRIM14	DPP7	POLE4
KIF22	VWCE	DSG2	PPL
LALBA	CCKBR	ETS1	PPP5C
LBP	DNMT1	FADS1	PRAF2
LTB	DNMT3B	FAR1	PRKCI
LTF	HOTAIR	FGF7	PTPRJ
MCM10	NR1I2	FMNL2	RAB23

MCPH1	RPS6KA5	GNA13	RAB27B
METTL7A	ABHD16A	HSD17B12	RAB34
MMP16	AGTR1	HSDL1	RAB5C
RAB6A	ACTR1A	FGF2	MAP2K1
RAI14	ADSS2	FGFR1	MAP2K4
RCN2	ANLN	FLT3	MAPK3
RCOR1	ARHGDIA	GALNT13	MCL1
RHEB	ARL2	GALNT7	MGAT4A
RHOA	ASXL2	GFM1	mir-9
RIPK1	ATF6	GFPT1	MLLT11
SATB1	ATG9A	GNL3L	MRPL20
SCAMP1	BCL2L2	GOLGA5	MSH2
SDCBP	BDNF	GOLPH3L	NAA15
SH3BP4	BMI1	GPAM	NAPG
SLA	C17orf80	GRB10	NFIA
SLC30A1	C2orf74	GRB2	NIPAL2
SMAD1	CA12	GSTM4	NOTCH2
SMAD2	CACNA2D1	GTF2H1	NPR3
SNAP29	CADM1	HACE1	NT5DC1
SOCS1	CAPRIN1	HARS1	OGT
SPI1	CARD8	HDHD2	OMA1
SYNE2	CCND3	HERC6	OSGEPL1
TAB2	CCNE1	HPF1	PAFAH1B2
TACSTD2	CCNF	HSDL2	PANX1
TBCA	CDC14A	HSP90B1	PDCD4
TCF7L2	CDC14B	HSPA1A/HSPA1B	PDCD6IP
TM6SF1	CDK5RAP1	HYAL3	PHKB
TNFRSF10A	CENPJ	IGF1	PHLDB2
TP53INP1	CEP63	IGF2R	PISD
TRAM1	CFL2	ITGA2	PLAG1

TRIM32	CHEK1	JUN	PMS1
TRIP13	CHORDC1	JUN/JUNB/JUND	PNN
TXNDC12	CLDN12	KCNN4	PNP
TXNRD1	CREBL2	KIF23	PPIF
UBE2J1	CRHBP	KITLG	PPP2R5C
UFL1	CSHL1	KPNA3	PRIMPOL
VAMP3	DIPK1A	LAMC1	PSAT1
WDFY1	DMTF1	LAMTOR3	PURA
WEE1	DNAJB4	LAMTOR5	PWWP2A
ABCF2	ECHDC1	LDAH	RAB21
ABHD10	EGFR	LUZP1	RAB30
ACP2	FAM122C	LYPLA2	RAB9B
RAD51C	UGDH	RBL2	CTNND1
RAF1	UGP2	RUNX1	TSPAN3
RARS1	UTP15	S1PR1	DYRK1A
RECK	VPS45	STAT3	LAMC2
RFT1	VTI1B	TGFBR2	SET
RHOT1	WIPF1	TLR7	SIRT1
RIDA	WNT3A	TNF	ACTA2
RNASEL	WT1	TP63	BTG2
RTN4	YIF1B	ZBTB7A	SGK3/SGK3
SEC24A	ZNF559	AKT1	FAM3C
SERPINE2	ZNF622	CDC42	FAS
SHOC2	ZYX	CORO2B	FASLG
SKAP2	APP	DDIT3	GLCCI1
SLC12A2	ARID4B	DDX43	GSK3B
SLC16A3	BAMBI	FOXO1	IL6R
SLC25A22	BCL2L11	TWIST1	JAG1
SLC35A1	BMPR2	CCN2	LRRFIP1
SLC35B3	BNIP2	HIF1A	MARCKS

SLC38A5	CAMTA1	Ccl9	MTAP
SLC7A1	CDKN1A	IL6	NFIB
SPTLC1	CREB1	TLR3	PELI1
SQSTM1	CRIM1	DHFR	PIK3R1
SRPRA	E2F1	DTL	PRRG4
SRPRB	E2F2	ZEB1	RP2
TIA1	ESR1	ZEB2	SERPINB5
TMEM109	HBP1	E2F6	SESN1
TMEM189	HIPK3	ERBB4	SLC12A1
TMEM251	ITCH	PLAU	SLC16A10
TMEM43	JAK1	PTK2	SOCS5
TNFSF9	MAP3K12	RPS6KB2	SOD3
TOMM34	MEF2D	ANXA1	SOX5
TPI1	MICA	HOXA7	SPRY2
TPPP3	MMP3	HOXB8	TCF21
TRMT13	MYLIP	HOXC8	TIMP3
TXN2	NCOA3	HOXD8	TPM1
UBE2S	PAK5	IKBKB	ATF4
UBE4A	PKD2	KRT5	GPD1
UCP2	PPARG	S100A9	ING4
MAN1A1	PTEN	ACVR1	POU4F2
SCN3A	MEF2C	MLF1	IDH1
BMP7	MMP13	MYBL2	KRT85
MAX	ODC1	NASP	LMNB2
PPARA	PDPK1	NAV3	LRRC8C
SRF	PEX7	PPIC	LTN1
BBC3	PHB	PPM1D	MAP4K4
BNIP3L	PKMYT1	RERE	MAT2A
CDKN1B	PXN	SHROOM2	MBNL1
CDKN1C	RXRA	SP1	MPDU1

DDIT4	SRM	SPARC	NCEH1
DIRAS3	ST14	SRSF10	NCL
FOS	THRB	TCL1A	NEUROD1
FOXO3	ZBTB10	TDG	NT5C3A
ICAM1	AR	TET1	NUCB1
KIT	ACVR2A	TGFB3	NUFIP2
PPP2R2A	ARPC3	TNFAIP3	P4HA2
PTPRM	BACE1	TRIM9	PEX11B
SOD2	CAV2	TUBB2A	PGM1
TBK1	CD276	YY1	POGLUT3
2700046G09Rik	CNOT8	ZFP36L1	PPP3R1
ATAT1	COL15A1	ADPGK	PRPF40A
FBXO32	COL1A1	ANPEP	PTGFRN
GATD3A/GATD3B	COL3A1	AP2A1	PTPA
HES1	COL4A1	ATP2A2	PTPRK
LMNB1	COL4A2	ATRX	PTRH1
MDH2	COL5A2	BECN1	RAD23B
NOTCH1	COL5A3	CDCP1	RBMS1
TRIM63	DCP2	CEP72	SEC23A
EPHA2	DUSP2	CHD1	SEC62
EZH2	FBN1	CNOT9	SLC12A4
HPGD	FRAT2	CPNE8	SLC38A2
MAP2	GAS7	DOCK7	SLC4A10
PHF6	GMFB	ELMOD2	SLC4A7
ADORA2B	GPR37	FRG1	SLC7A11
CTNNBIP1	HMGN3	FXR2	SLC9A3R2
CYP1B1	INSIG1	GALNT1	STRN
FBXW7	KCTD3	GNAI2	STX1A
GCA	LOXL2	GPD2	STX7
IFI16	MAPRE2	HNRNPM	SYT4

THEM4	CNTN4		
TMCO1	CDKN2AIP		
TMED10	VSNL1		
TMED2	ONECUT1		
TMED3	AGO1		
TMED7	CCNE2		
TMEM41B	RUNX3		
TMEM59	LCOR		
TMEM87A	NTRK3		
TNFAIP2	BCL2L12		
TNFRSF10B	MAD2L1		
TNRC6A	ENPP6		
UAP1	IKZF1		
WNT5A	ITGA5		
CASR	MAPRE1		
FOXP3	OSBPL2		
LATS2	OSBPL8		
PDGFB	PCGF1		
SATB2	RFFL		
STK40			
GNAO1			
LIMK1			
NANOG			
NR5A2			
PUM2			
HSPB6			
PTPN11			
GRM3			
HOXA11			
MTDH			

AXIN2			
CCN5			
DLL1			
E2F5			
FOXP1			
HDAC1			
MYCN			
MAP3K8			
TEAD1			

Supplementary Table 4. Unique mRNA targets from miRNA sequencing results

**PLT unique targets**	**FBS unique targets**
**Gene ID**	**Gene ID**
APC	AACS
JAK2	ADAM17
SLC6A4	AKT3
HSF2	ALDOA
PIAS3	ANK2
NR4A2	ANXA11
SPRY1	AP3M2
ACVR1B	ATP11A
CASP8AP2	ATP1A2
EFNA3	BACH2
FGFRL1	BCKDK
ISCU	CCNG1
MNT	CD320
NPTX1	CERS6
PTPN1	CLDN18
RAD52	CS
SDHD	DSTYK

TP53I11	EGLN3
RBMXL1	FAM117B
ADCY6	FOXJ3
AHCYL1	FUNDC2
AQP5	G6PC3
CELSR2	GALNT10
HTR1B	GPX7
MITF	HJV
MYRIP	MAPK11
ODF2	MEP1A
RYK	NCAM1
	NDRG3
	NFATC1
	NFATC2IP
	NUMBL
	OSMR
	PALM
	RAB11FIP1
	RAB6B
	RABIF
	SLC35A4
	TBX19
	TMEM50B
	TPD52L2
	TRIB1
	TRPV6
	TTYH3
	UBAP2
	XPO6
	Spry1

	ABCB1
	HGS
	SCRIB
	ABCG2
	CD44
